# A Novel Synthesis Routine for Woodwardite and Its Affinity towards Light (La, Ce, Nd) and Heavy (Gd and Y) Rare Earth Elements

**DOI:** 10.3390/ma11010130

**Published:** 2018-01-14

**Authors:** Sirio Consani, Tonci Balić-Žunić, Anna Maria Cardinale, Walter Sgroi, Gabriele Giuli, Cristina Carbone

**Affiliations:** 1 Dipartimento di Scienze della Terra dell’Ambiente e della Vita (DISTAV), University of Genova, Corso Europa 26, Genova 16132, Italy; carbone@dipteris.unige.it; 2 Department of Geosciences and Natural Resource Management, Copenhagen University, Øster Voldgade 10, Copenhagen K 1350, Denmark; toncib@ign.ku.dk; 3 Dipartimento di Chimica e Chimica Industriale (DCCI), University of Genova, Via Dodecaneso 31, Genova 16146, Italy; cardinal@chimica.unige.it (C.A.); walter.sgroi@unige.it (S.W.); 4 School of Science and Technology-Geology division, University of Camerino, Camerino 62032, Italy; gabriele.giuli@unicam.it

**Keywords:** woodwardite, hydrotalcite supergroup, layered double hydroxides, rare earth elements, synthesis

## Abstract

A synthetic Cu-Al-SO_4_ layered double hydroxide (LDH), analogue to the mineral woodwardite [Cu_1−x_Al_x_(SO_4_)_x/2_(OH)_2_·nH_2_O], with x < 0.5 and n ≤ 3x/2, was synthesised by adding a solution of Cu and Al sulphates to a solution with NaOH. The pH values were kept constant at 8.0 and 10.0 by a continuous addition of NaOH. The material obtained had poor crystallinity, turbostratic structure, and consisted of nanoscopic crystallites. The analyses performed in order to characterise the obtained materials (X-ray diffraction (XRD), thermogravimetry (TG), and Fourier Transform Infra-Red (FTIR) spectroscopy) showed that the Cu-Al-SO_4_ LDH is very similar to woodwardite, although it has a smaller layer spacing, presumably due to a lesser water content than in natural samples. The synthesis was performed by adding light rare earth elements (LREEs) (La, Ce, and Nd) and heavy rare earth elements (HREEs) (Gd and Y) in order to test the affinity of the Cu-Al-SO_4_ LDH to the incorporation of REEs. The concentration of rare earth elements (REEs) in the solid fraction was in the range of 3.5–8 wt %. The results showed a good affinity for HREE and Nd, especially for materials synthesised at pH 10.0, whereas the affinities for Ce and La were much lower or non-existent. The thermal decomposition of the REE-doped materials generates a mixture of Cu, Al, and REE oxides, making them interesting as precursors in REE oxide synthesis.

## 1. Introduction

Rare earth elements (REEs) is the common term used to refer to the elements from lanthanum to lutetium (atomic numbers 57 to 71). Often, scandium and yttrium are also grouped with REEs due to their similar chemical behaviour. This group of elements is subsequently divided in light REEs (LREEs; from La to Eu) and heavy REEs (HREEs; from Gd to Lu, plus Y). Due to the difference of the ionic radii between the two groups, mineral structures tend to preferentially incorporate elements belonging to LREEs or HREEs. REEs have received increasing attention in the past decade due to their wide applications (catalysts, magnets, alloys, ceramics, electronics, and other; [1]).

Acid mine drainage (AMD), due to its high acidity, can act as a natural “heap leaching” process, mobilising important concentrations of REEs and generating precipitates anomalously enriched in REEs [2]. In such environments, REEs in solution can form both cations in the form of [REE(H_2_O)_6_]^3+^ and anions in the form of [REE(SO_4_)_2_]^−^ [3], thus interacting in different ways with minerals. In the abandoned Cu mine of Libiola (Northwest Italy), where the generation of AMD is widespread, woodwardite’s (Cu_1−x_Al_x_(SO_4_)_x/2_(OH)_2_·nH_2_O) presence is linked with high concentrations or REEs, such as Y (up to 600 mg kg^−1^), Ce and Nd (up to 200 mg kg^−1^), and Gd and Dy (up to 100 mg kg^−1^).

Woodwardite belongs to the hydrotalcite supergroup [4], and the synthetic analogues of these minerals are often called layered double hydroxides (LDHs). This mineral mainly forms crusts, stalagmites, or nanoscopic precipitates in copper mining areas generated by waters with high metal content and circumneutral pH [5,6,7,8,9]. The crystal structure of the minerals of the hydrotalcite supergroup is composed of brucite-type layers in which a trivalent cation partially substitutes a divalent cation, generating a net positive charge balanced by an anionic species in the interlayer, giving a general formula M^2+^_1−x_M^3+^_x_(A^z−^)_x/z_(OH)_2_·nH_2_O. Minerals of the hydrotalcite supergroup comprise different polytypes with varying periodicity along the *c* axis or differing in the order/disorder of cations and/or anions [10,11,12,13,14,15,16]. As a consequence of the fact that REEs can form both anions and cations in solution in AMD environments, these elements might be hosted inside the crystal structure of woodwardite in the octahedral layers or in the interspace between them. The minerals of the hydrotalcite supergroup and, even more, their synthetic analogues, received, in recent decades, increasing attention as “anionic clays” due to their capacity to easily exchange the interlayer anions [17,18] and to their flexible structure. Synthetic LDHs can be obtained using relatively simple routines of synthesis and display a wide range of bivalent and trivalent cations with different ratios [19]. Such features make LDHs promising materials for industrial applications in many fields: catalysis, ion exchange/adsorption, pharmaceuticals, photochemistry, and electrochemistry [20]. Natural LDHs normally display a M^2+^/M^3+^ ratio of 2:1 or 3:1, although synthetic compounds were synthesised with different ratios.

The affinity of LDH compounds towards REEs was investigated by several authors, often with the intercalation of organic complexes inside the interlayer [21,22,23]. In any case, the specific capacity of woodwardite to scavenge REEs from solutions is suggested by the work of Kameda et al. [24]. In that work, a Cu-Al LDH intercalated with ethylenediaminetetraacetate (EDTA) was used to scavenge Sc, La, and Y from solution, and the results suggested that synthetic Cu-Al LDH was able to also scavenge Y in its pure form. Apart from this work, little data are available in the literature on REE uptake by Cu-Al LDH.

In order to explore the incorporation of REEs in the woodwardite structure, the synthesis and doping with REEs of this mineral has been performed. The method used for the synthesis was co-precipitation at constant pH, modifying the method proposed by [25,26], thus obtaining a new synthesis routine. The tested REEs were chosen among light REEs (La, Ce, and Nd) as well as heavy REEs (Gd and Y), in order to investigate possible preferential incorporation on the basis of the ionic radius. The synthesis was carried out at the two different pH values: 8.0 and 10.0, as these values are reported for routine synthesis of LDHs and allowed the determination of an eventual role of pH in REE incorporation. Moreover, values of pH around 8.0 are close to the ones observed in natural occurrences of woodwardite, whereas pH 10.0 looks more promising for REE incorporation, because the low supersaturation method leads to more disordered hydrotalcites, favouring REE incorporation [27].

## 2. Materials and Methods

### 2.1. Synthesis of Woodwardite and REEs Addition

The synthetic analogue of woodwardite, a Cu-Al-SO_4_ LDH, was prepared by a dropwise addition of 200 mL of a concentrated Cu-Al sulphate solution, with a Cu:Al molar ratio of 3:1, to 200 mL of deionised water. The Cu:Al molar ratio was chosen on the basis of the typical Cu:Al ratio in natural samples of woodwardite. The reagents used in the synthesis were of scientific grade.

The equipment to carry on the synthesis reaction was set up in order to carefully and continuously control and adjust both the volume of the Cu-Al sulphate solution added and the pH. The solution addition was via a graduated burette, the pH condition was monitored by means of a pHmeter pH/ORP/ISE Single Channel Benchtop Meter-HI3221 (HANNA Instruments, Woonsocket, RI, USA) equipped with a combined pH electrode. The pH was kept constant at the required value by adding, when necessary, a 0.5 M solution of NaOH.

The precipitates were aged with their mother liquor for one week at 50 °C, then separated by filtering on a glass funnel and filter paper, and washed abundantly with deionised water. The final product was a blue powder. According to the nomenclature proposed by Mills et al. (2012) [4] for synthetic LDH, synthetic woodwardite samples at different pH values will hereafter be referred as LDH 3CuAl·SO_4_-pH8 and LDH 3CuAl·SO_4_-pH10, respectively. 

The REE-doped synthetic woodwardite samples were also prepared at pH 8.0 and 10.0 with the same method, substituting part of Al with REEs (La, Ce, Nd, Gd, and Y), so that the Cu:Al:REEs molar ratio was 3:0.8:0.2. The values of pH were kept constant at 8.1 ± 0.2 or 10.1 ± 0.2 by adding a 1 M solution of NaOH. The REE salts used to prepare the starting solutions were Y(NO_3_)_3_, La(NO_3_)_3_, Ce_2_(SO_4_)_3_, a mixture of Nd carbonates, and Gd sulphate. For simplification, the REE-doped samples will be labelled hereafter indicating LDH followed by the REE followed by the pH value. Therefore, the sample of synthetic woodwardite doped with La at pH 8 will be indicated as LDH-La-8, the sample doped with Ce at pH 10 will be indicated as LDH-Ce-10, and so on.

### 2.2. Characterisation Methods

All samples were analysed by powder X-Ray diffraction (PXRD) using a AXS D8 Advance diffractometer (Bruker, Billerica, MA, USA) equipped with the primary-beam Ge_111_ monochromator, Cu tube (Kα1 wavelength: 1.5406 Å) and the silicon-strip detector (LynxEye, Bruker, Billerica, MA, USA) at the XRD laboratory of the Department of Geosciences and Natural Resource Management of the University of Copenhagen. The patterns were collected in reflection Bragg-Brentano geometry between 2° and 90° 2θ with a step of 0.02° and measuring time of 4 s/step. The samples were mounted in rotating sample holders with the sample thickness of 2 mm. Crystal lattice parameters were obtained fitting 001, 002, and 110 reflections of woodwardite-like phases performed using the software TOPAS (V4.1, Bruker, Billerica, MA, USA) and Si as an internal standard.

The chemical analyses were performed by inductively-coupled plasma mass spectrometry (ICP-MS) after dissolution of the samples in concentrated nitric acid solution. To verify the possibility of carbonate presence in the interlayer (from atmospheric CO_2_) Fourier Transform Infra-Red (FTIR) spectroscopy was performed using a Spectrum 65 FT-IR Spectrometer (PerkinElmer, Waltham, MA, USA) equipped with a KBr beamsplitter and a DTGS detector by use of an ATR accessory with a diamond crystal. All spectra were recorded from 4000 cm^−1^ to 700 cm^−1^. The samples, enclosed in alumina crucibles, were characterised also by means of thermogravimetry (TG). The TG analyses were carried out in alumina crucibles under argon flux, at a heating rate of 5 K min^−1^; the instrument used was a model H/LABSYSEVO-1A SETARAM apparatus. After the TG-DTA the samples were collected and analysed by PXRD with a Philips PW1140 diffractometer (PANalytical, Almelo, The Netherlands) equipped with CoKα radiation (current 20 mA, voltage 40 kV) at the laboratory of the University of Genova. Samples were ground with an agate mortar and pestle, and mounted on zero-background silicon plates. Each sample was scanned between 5° and 80° 2θ at a scan rate of 5°/min.

SEM (scanning electron microscope) observations were carried out with a Vega 3 LMU scanning electron microscope (Tescan, Brno, Czech Republic) equipped with an energy dispersive spectroscopy (EDS) EDAX Apollo X SDD (AMETEK, Philadelphia, PA, USA), at 20 kV accelerating voltage, 1.2 nA beam current, and 5–10 μm beam diameter. In order to prevent damage to the coated surface of the samples, counting times were set at 60 s. Calibration was accomplished with a set of natural standards for the elements reported in parenthesis: omphacite (Na, Mg, Al, Si, and Ca); scapolite (Cl); barite (Ba), and ilmenite (Fe, Mn) and for the other elements with the synthetic standards supplied by MAC (MAC, St Ives, Cambridgeshire, UK).

X-ray absorption spectroscopy (XAS) at the S k-edge was also performed. The measurements were carried out at beamline 4–3 of the Stanford Synchrotron Radiation Lightsource (SSRL) storage ring (Stanford, CA, USA). The theoretical resolution of the beamline monochromator with a cryogenically-cooled Si (111) double crystal is 0.36 eV at the S k-edge energy (2472.0 eV). Beam size and flux were 2 × 10 mm and 10^12^ photons/s higher harmonics were rejected by two Si mirrors operating in total reflection. Instrumental errors due to temperature variations or other instabilities in the optic elements of the beamline were within the precision limit of 0.05 eV.

X-ray Absorption Near Edge Structure (XANES) spectra were recorded in fluorescence mode using a Si drift detector (HTA 4-element, Hitachi, Tokyo, Japan) mounted horizontally at a 90° scattering angle to the incident X-ray beam. The Total Fluorescence Yield (TFY) spectra were obtained by monitoring the diode current while the incident energy was scanned across the sulphur edge. The total acquisition time for each XANES spectrum was 1 h. Four spectra have been collected and averaged. Energy calibration was achieved by setting the k-edge of native sulphur to 2472.0 eV. Standard data-reduction procedures were applied to extract the XANES by subtracting a linear background before the edge and normalising the jump height to one at the high-energy part of the spectrum.

## 3. Results and Discussion

### 3.1. Characterisation of the Synthesis Product

**Figure 1:**
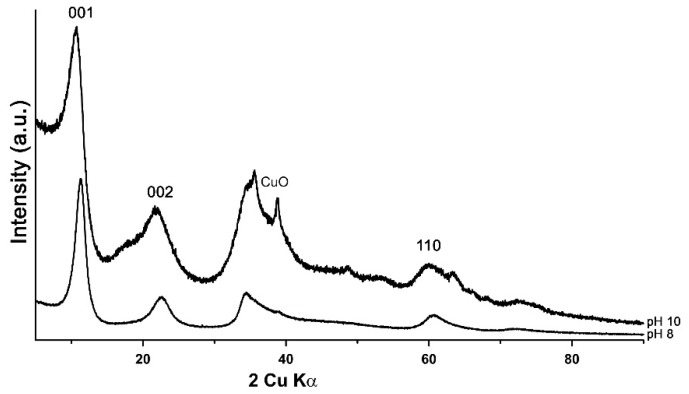
PXRD patterns of LDH 3CuAl·SO_4_ at pH 8 (below) and pH 10 (above).

The PXRD diagrams of LDH 3CuAl·SO_4_-pH8 and LDH 3CuAl·SO_4_-pH10 (Figure 1) confirmed the success of the synthesis routines, as the synthetic compounds had a powder XRD pattern comparable to woodwardite. In the sample synthesised at pH 10.0, admixed with the LDH compound, CuO reflections were detected together with a broad maximum at around 17° 2θ, attributed to poorly-crystalline Cu(OH)_2_. The main difference between the XRD patterns of our synthetic LDHs samples and natural woodwardite is in the interplanar spacing of the structural layers, which varies from 9.1 to 8.9 Å in natural samples [4,5], whereas the values measured in our samples were lower (7.90 and 8.17 Å, respectively). This difference might be due to a lesser hydration or to a partial substitution of sulphate by smaller anions (such as carbonate) in synthetic samples, due to the synthesis method [28]. The amount of structural water, measured by TG, indeed turned out to be significantly lower in synthetic samples, whereas the FTIR spectra show almost negligible contents of carbonate (see below), so our results suggest that the difference is primarily due to lesser hydration.

**Figure 2:**
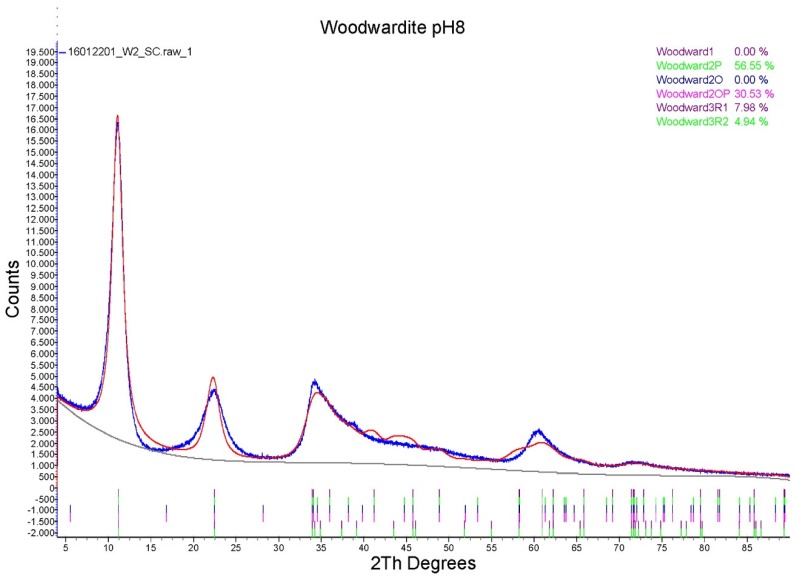
Rietveld refinement results (red line) of the LDH 3CuAl·SO_4_ at pH 8 PXRD diagram (blue line) with different polytypes.

The features of the powder diffraction pattern (Figure 1) show that the structure of synthetic samples is a turbostratic one [29], suffering from high amounts of random stacking faults. This is illustrated well by reflection at around 34° 2θ, representing an overlap of the 009 reflection with a sequence of hkl reflections of various possible polytypes producing a long high-angle tail. Such disorder is very common in synthetic LDH [30]. To verify the structural similarity of the synthetic product to woodwardite, a fitting of a mixture of the lowest six possible polytypes [10] with structures based on the natural woodwardite [31] was attempted (Figure 2). Although the fitting gives a relatively good match of the observed and calculated intensities of the reflections, confirming the structural model, the fit of the profiles is poor, showing that the diffraction features of this highly disordered material with very small crystallite size (estimated 3 to 5 nm) cannot be fitted by a standard Rietveld refinement.

The chemical analyses (Table 1) are in good agreement with those reported by [5] for natural samples, although slightly lower in Al. This fact could be due to the presence, in natural samples, of small amounts of amorphous Al colloids, as also observed in other works [7]. The empirical formula obtained from chemical analyses (Table 2) was calculated on the basis of 1 cation per formula unit, with sulphate calculated according with Al content. The Cu/Al ratio of the 3CuAl·SO_4_-pH8 sample is in good agreement with the molar ratio of the solution, while, on the contrary, the 3CuAl·SO_4_-pH10 sample showed a marked enrichment in Cu in the solid fraction, which may be due to the formation of CuO and Cu(OH)_2_. In general, for the synthetic samples, the x parameter, defined as Al/Al + Cu, lies inside, or is at least very near to the range of 0.23–0.67 reported by [32] for natural and synthetic samples of hydrowoodwardite, the more hydrated form of woodwardite.

**Figure 3:**
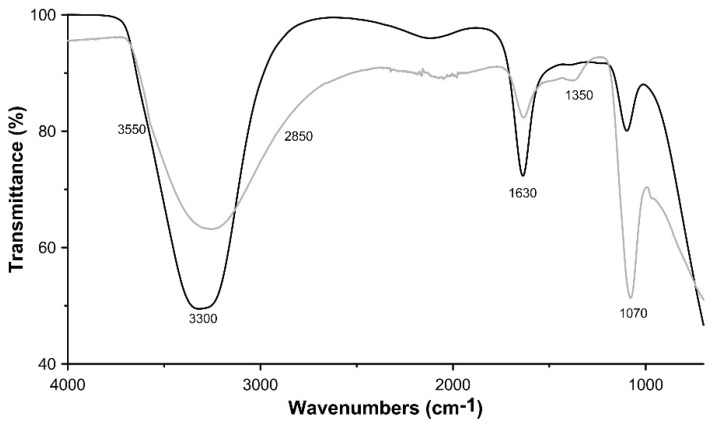
FTIR spectra of LDH 3CuAl·SO_4_ at pH 8 (black line) and pH 10 (grey line).

In Figure 3 the IR spectra of the two synthetic samples are reported. The two spectra are comparable to those of natural woodwardite, or to those of other members of the woodwardite group [5,31,32,33]. Both spectra showed a broad and intense absorption band ranging from 3680 to 2850 cm^−1^ due to the OH stretching mode of hydroxide groups (maximum at 3300 cm^−1^) and of interlayer water molecules (around 2850 cm^−1^), respectively. The position of this band should be dependent on the nature of the hydroxy-layer cation, as its electronegativity should modify the electron density on the O–H bond (M–OH). However, the extreme broadness of this band, owing to hydrogen bonding, precludes any meaningful discussion. A weak shoulder recorded around 3550 cm^−1^ in the pH 10.0 synthetic sample has been ascribed to the OH stretching mode of hydroxide group hydrogen-bonded to interlayer carbonate anions. The presence of carbonate, although in very small concentration, is also confirmed by the absorption band at 1350 cm^−1^ (C–O asymmetric stretching; [5]) and is due to the reaction with atmospheric CO_2_ at basic pH. The peak located at 1630 cm^−1^ is assigned to OH bending, whereas the strong absorption band at 1070 cm^−1^ is due to sulphate ν_4_ bending mode.

**Figure 4:**
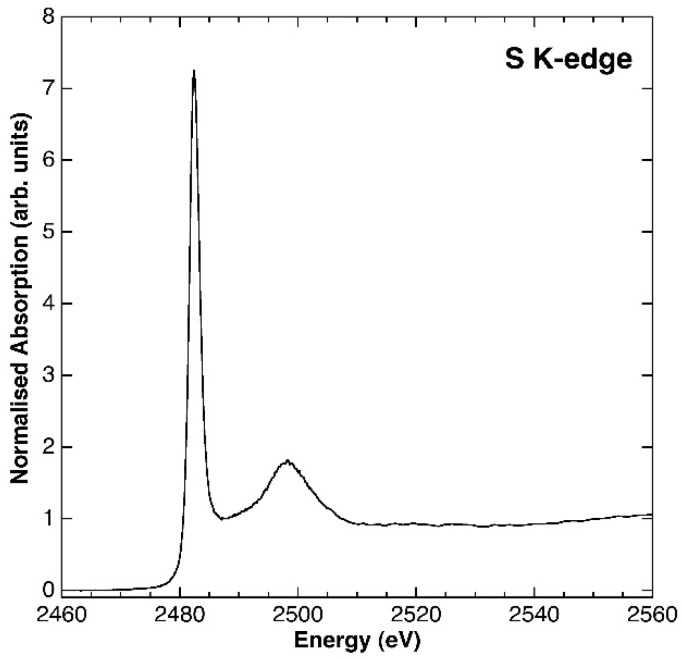
XAS spectrum at the S k-edge of the synthetic woodwardite sample.

The XANES spectrum allowed excluding other oxidation states for S, which could also influence the layer spacing (Figure 4). The spectrum displays a well resolved white line centred at 2482.3 eV. The edge energy, as determined by the derivative spectrum (at 2481.7 eV), compares well with those of sulphate model compounds published in the literature (ranging from 2482.5 to 2482.9; [34,35]. As no contribution has been detected at lower energies, possibly related to S^0^ (edge energy = 2472.86), sulphide (edge energies ranging from 2470.4 to 2473.8) or sulphite (edge energy = 2478.62), their presence can be excluded.

**Figure 5:**
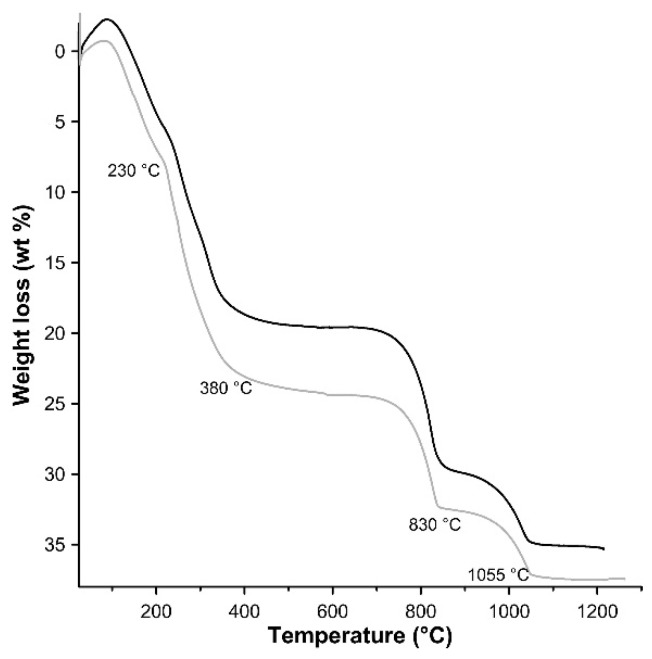
TG patterns of LDH 3CuAl·SO_4_ at pH 8 (black line) and pH 10 (grey line).

TG analysis was performed on the 3CuAl·SO_4_-pH8 and 3CuAl·SO_4_-pH10 samples (Figure 5), which were subjected to a weight loss in the range of 35–37 wt %. The first mass decrease (5 and 8 wt %, respectively) occurs from room temperature to ≈230 °C, whereas the second weight loss (14 and 16 wt %, respectively) takes place up to ≈380 °C. These weigh losses are attributed to interlayer water and to dehydration of the hydroxide layers. The residual mass reduction takes place in two steps at about 830 °C (desulphurisation) and 1055 °C (Cu^2+^ to Cu^+^ reduction). In general, the 3CuAl·SO_4_-pH8 sample had a lower H_2_O content than the 3CuAl·SO_4_-pH10 sample, most probably explaining its somewhat smaller layer spacing observed with XRD. The water content found with TG for the two samples was slightly lower than the total water content (for the entire range of T) of 25.1 wt % observed in natural samples of similar compounds [31].

**Figure 6:**
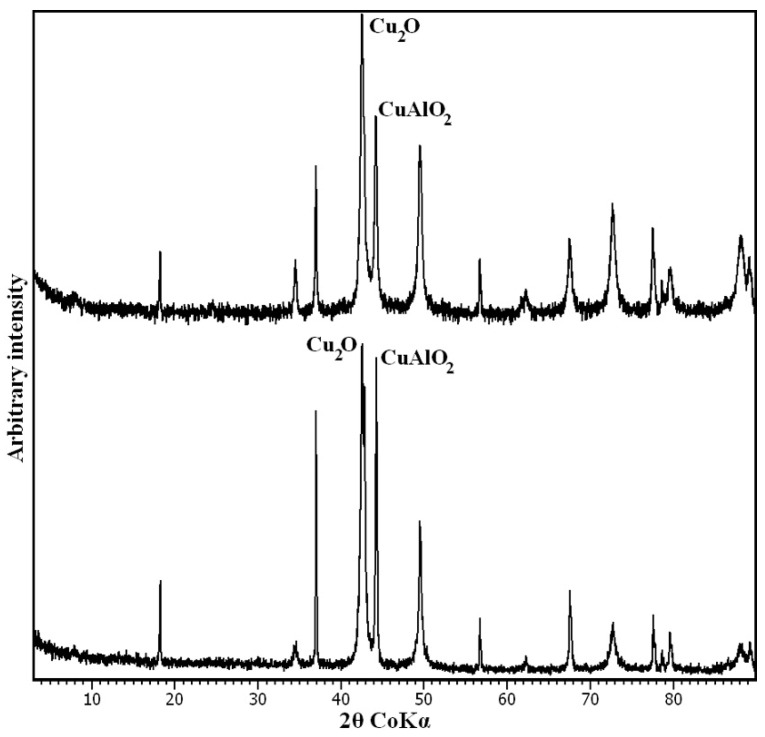
PXRD pattern of LDH 3CuAl·SO_4_-pH8 (below) and LDH 3CuAl·SO_4_-pH10 (above) after heating at 1400 °C. Only the main maximum for every phase is labelled.

After TG analysis, the residuals were analysed by PXRD. The decomposition products were two oxides of Cu^+^, Cu_2_O and CuAlO_2_. However, the slight asymmetry of the main reflection of Cu_2_O, located at 36.4° 2θ (Figure 6), also suggests the presence of small amounts of Cu^2+^Al_2_O_4_. This phase is assumed to be the precursor, along with CuO, of the final Cu^+^ oxides. The formation of these mixtures of single and double oxides is typical of LDH thermal decomposition [28,36].

### 3.2. REE-Doped Samples

**Figure 7:**
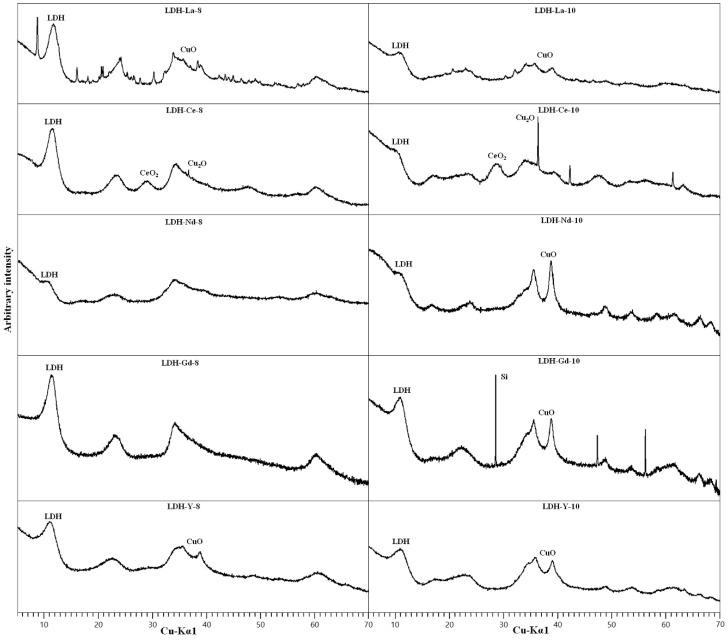
PXRD patterns of REEs-doped samples. Only the main maximum for every phase is labelled.

All the synthetic samples with REEs had an LDH structure as shown by PXRD (Figure 7). By-products CuO and Cu(OH)_2_ were detected in samples synthesised at pH 10, similarly to what was observed for non-doped samples. The diffraction pattern of the LDH phase again shows features of a structure highly disordered by layer-stacking faults. The chemical analyses results and the calculated LDH formulas (Table 1 and Table 2) are comparable to that of non-doped samples. The formulas were calculated subtracting from the Cu amount found with chemical analyses the amount of Cu estimated to be in by-product oxides obtained through approximate Rietveld refinement. The REE content is between 3.5 and 8.0 wt %, showing that the solid phase has a strong affinity towards these elements, especially for samples synthesised at pH 10, which show a higher concentration of REEs (Table 1). The calculated Cu/Al molar ratio in LDH is usually near to 3:0.8 but, in general, a little lower. This might suggest that a part of the starting Cu remained in solution, but could also be a result of the errors in the estimation of the contents of additional phases. The only exception is LDH-Nd-10, which shows a very high Cu:Al ratio.

The REE-doped samples were also characterised by means of FTIR and TG, giving the same results of the synthetic pure products. In particular, the only anions found with FTIR were sulphate and small amounts of carbonate, whereas the water content obtained with TG was, again, slightly higher for samples synthesised at pH 10.0.

**Figure 8:**
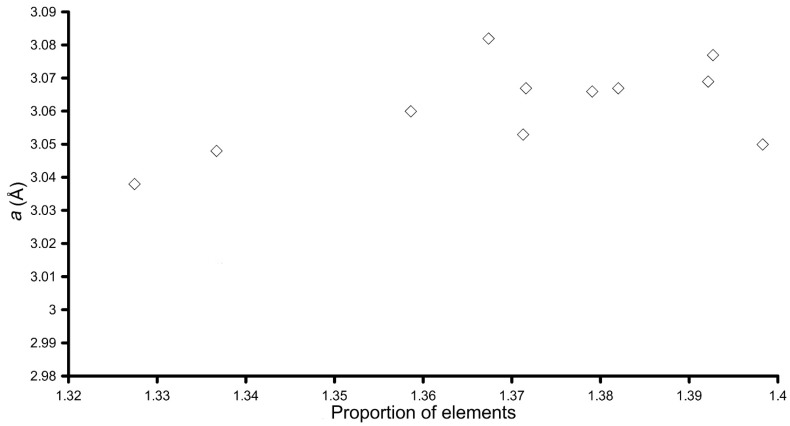
Plot of the proportion of elements vs. *a* for the LDH synthetic samples.

The crystal lattice parameters obtained from fitting of the 001, 002, and 110 reflections are reported in Table 2. The *a* parameter is very close to the one observed for the pure synthetic woodwardite, but some observations could still be done. It should be noted that, for samples synthesised at pH 10.0, its determination is less accurate and difficult, and in one case (Nd-doped sample), even impossible because of overlap with the maxima of other phases. In any case, plotting the *a* parameter (from Table 2) vs. the proportion of elements, calculated as two times the atoms per formula unit (apfu) of the cations (obtained by dividing by a factor of 8 the formulas reported in Table 2) times their ionic radii of each element obtained by [37] (Figure 8), a correlation between the presence of REE in the LDH structure and *a* could be seen. In fact, *a* tended to increase according to the proportions of the elements, which also tended to increase in the presence of REEs due to their larger ionic radii. This fact, taking into account uncertainties of the *a* parameter determination due to broadening of reflections, and to the turbostratic nature of the material, strongly suggest that REEs are present as trivalent cations inside the hydroxyl layer, substituting for Al.

To check what the diffraction characteristics say about the possible incorporation site of REE in the woodwardite structure, an approximate Rietveld refinement of the sample LDH-Gd-8 that did not show any additional phases on the XRD diagram was made again using a mixture of the possible first six polytypes, placing a potential REE site mid-way between the hydroxide layers at a position between the two S sites belonging to the “up” and “down” orientation of the sulphate tetrahedron. At the same time, the Cu occupancy of the cation site in the hydroxide layer was allowed to change in refinement up to the value possible from the chemical formula. The result was a zero occupancy of the interlayer site and a maximum increase of the occupancy of the cation site inside the hydroxide layer, thus supporting the conclusion that REEs are incorporated in the structure by a substitution of Al in the hydroxide layers. Moreover, the presence of a large ion cation, such as REEs, in the interlayer would result in an increase of the observed d001 to values ≥11 Å, as observed in similar cases for the wermlandite-group minerals [4]. We are well aware, however, that Rietveld refinement on such samples is not completely reliable, and this indication is to be taken with extreme care.

It can be seen that the values of the *c* parameter differ significantly when determined from two reflections (001 and 002), testifying for the disorder and the small crystallite sizes that produce a variable shift of the diffraction maxima at different angles. Nevertheless, it can be observed that the layer spacing increases from pH 8.0 (range from 7.61 to 8.05 Å) to pH 10.0 (range from 7.92 to 8.39 Å) for the same type of composition. This fact could be explained with a higher hydration of the samples at higher pH. From this it can be concluded that pH influences the amount of water incorporated in the interlayer. 

As regards the composition of the REE-doped samples, LREE La and Ce showed relatively large amounts of additional phase at both pH 8.0 and pH 10.0. This could be connected with a more difficult incorporation of relatively large REE in the woodwardite structure. Similar problems with the incorporation of La in the LDH structure were reported by [27], who tried to synthesise La-doped Mg-Al-CO_3_ LDH, observing that La carbonate formation took place in the very early stages of the co-precipitation and in corroboration with its high ionic radius (1.302 Å in octahedral coordination) prevented the possible intercalation of larger lanthanum species in the LDH interlayer space. However, in our sample, other unidentified well crystalline species were present in the sample synthesised at pH 8.0. Due to the complex composition of the samples, we did not attempt to estimate the amount of La in the solid phase that belongs to the LDH and, therefore, we report in Table 2 the chemical composition without La.

**Figure 9:**
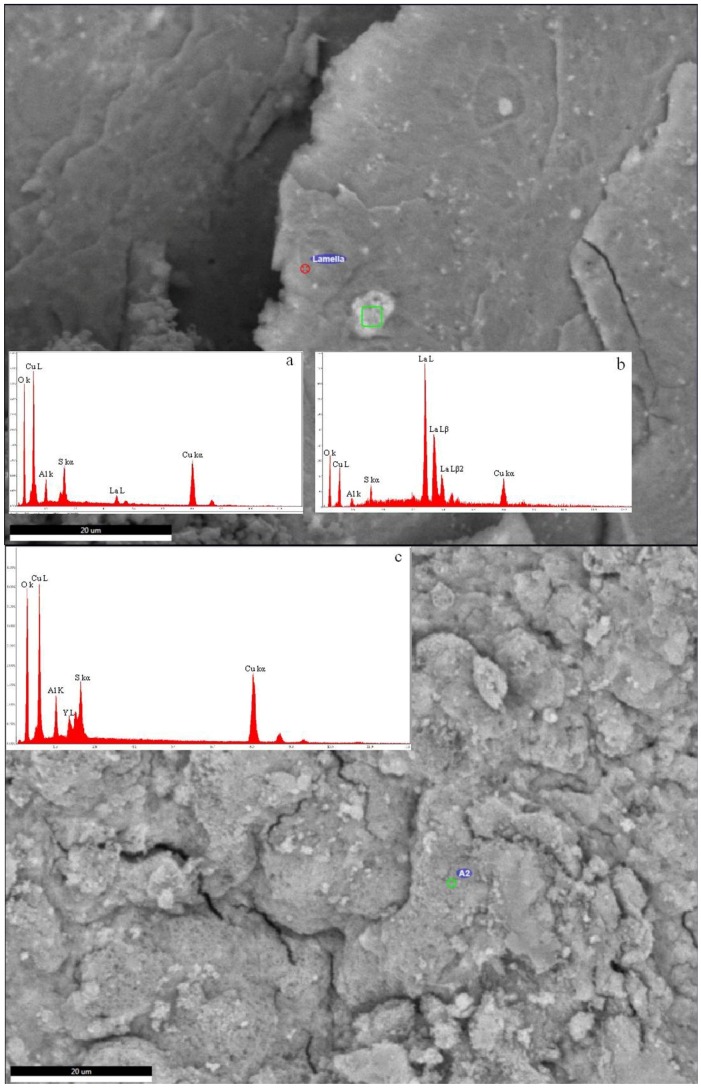
SEM image of LDH-La-10 (above) and LDH-Y-10 (below). In the insets the EDS semi-quantitative analyses are reported. (**a**) Analysis of the spot indicated in the LDH-Y-10 image; (**b**) analysis of the bright area in green in the LDH-La-10 image; and (**c**) analysis of the spot indicated in the LDH-La-10 image.

In Ce-doped samples a formation of CeO_2_ was detected together with Cu_2_O. Ce^3+^ was obviously at least partly oxidised by Cu^2+^ and precipitated as ceria, as already observed by [38]. An approximate Rietveld refinement of LDH-Ce-8, neglecting a very small amount of Cu_2_O, and assuming ceria as the only additional phase, showed that around 75% of Ce in the LDH-Ce-8 sample is contained in ceria, while the rest is assumed to be hosted in the LDH structure or adsorbed on the surface. In the LDH-Ce-10 sample with a very small proportion of LDH (Figure 9) the oxidation of Ce was most probably complete, as suggested by the more intense peaks of Cu_2_O, and we assume that no Ce was hosted in the LDH structure.

SEM observations showed patches of bright small aggregates with increased La concentration (inset c in Figure 9) dispersed in a matrix of a La-poorer Cu, Al, S, and O material (inset b in Figure 9), forming a homogeneous aggregate of nanoscopic crystals. Similar observations were seen in Ce samples. 

On the contrary, Nd-doped samples did not show any segregated phases, which means that Nd is homogeneously distributed in the solid phase. XRD diagrams of Nd-doped samples with very weak basal reflections of LDH and the appearance of the broad reflection at around 17° 2θ attributed to Cu(OH)_2_ already at pH 8.0, are the closest of all diagrams in resembling an amorphous phase, and this might be the reason why the Nd-doped samples incorporate the largest observed REE content. 

The samples doped with HREEs (Y and Gd) produce the best evolved diffraction patterns of LDH of all REE-doped samples. In the Y-doped sample additional CuO appears at pH 8.0, whereas the sample synthesised at pH 10.0 has both more CuO and the Cu(OH)_2_ diffraction maximum at 17° 2θ. The Gd-doped sample produces a pure LDH pattern at pH 8.0 and at pH 10.0 only CuO can be observed as an additional phase. The EDS semi-quantitative analyses confirmed the presence of these REEs, such as Y (Figure 9c), together with O, Cu, Al, and S. As these elements are the major constituents of synthetic woodwardite, and no other REE-containing phase has been observed by XRD or SEM, it can be concluded that REE (Y in this case) is entirely contained in this phase. 

**Figure 10:**
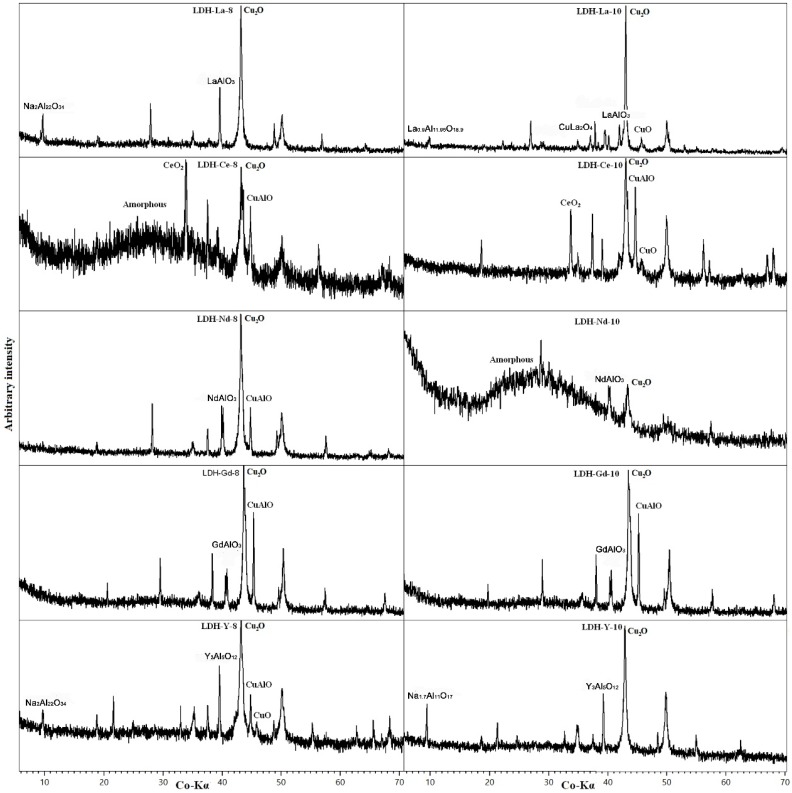
PXRD pattern of REE-doped samples after heating at 1400 °C. Only the main maximum for every phase is labelled.

The residuum after TG analysis was analysed by PXRD (Figure 10). Cu_2_O has been detected in all samples, deriving from LDH decomposition. CuO and CuAl_2_O_4_ can be interpreted, as for LDH 3CuAl·SO_4_ heated sample, as lower-temperature precursors of Cu^1+^ oxides. The fate of Al depends on the behaviour and content of REEs. La, Y, and Nd tend to form an oxide with Al (LaAlO_3_, YAlO_3_, and NdAlO_3_, respectively). In additin, La also formed a non-stoichiometric oxide with Al. If, as in LDH-Nd-8, Al content exceeds the REE content, it will form oxides with Cu. Na, which may be present from the synthesis routine in less-washed samples, forms NaAlO_2_. In Ce-doped samples where cerianite precipitates (quite stable above 1400 °C) all of the Al is hosted in CuAlO_2_. The broad amorphous background in LDH-Ce-8 and LDH-Nd-10 is produced by the glass support used to mount the sample.

## 4. Conclusions

The synthesis of a Cu-Al-SO_4_ LDH, an analogue of woodwardite, was achieved at two different pH values (8.0 and 10.0), both in a pure state and doped with LREEs or HREEs. The analyses showed that the synthesis produced a layered material phase structurally akin to natural woodwardite. The synthetic phase is poorly crystalline and highly turbostratically disordered with nano-sized crystallites. The phases synthesised at pH 10.0 have a slightly larger layer spacing and contained, in addition, CuO and Cu(OH)_2_, byproducts of the synthesis routine. Such byproducts might limit the effectiveness of the synthesis routine, rather than the applicability of the material. The formation of these phases is probably due to sudden changes of pH, and a method to avoid, or limit, their presence is currently being developed. REEs showed a relatively high affinity towards synthetic Cu-Al LDH, higher at pH 10.0 than at pH 8.0. Among LREEs, Nd was incorporated fully inside the structure of the semi-amorphous Cu-Al LDH, whereas Ce was partly oxidised to Ce^4+^ with only a smaller proportion incorporated in Cu-Al LDH. La formed separate well crystalline phases and was probably not incorporated in Cu-Al LDH. On the contrary, HREEs are totally incorporated inside the Cu-Al LDH. The analysis suggests that REEs are incorporated in Cu-Al LDH by replacing Al in the hydroxide layers. This also gives an explanation for the larger preference of the REE with a smaller ionic size for the incorporation in Cu-Al LDH. The thermal decomposition of the REE-doped Cu-Al LDH generates a mixture of pure and mixed Cu, Al, and REE oxides. It could, therefore, be used as a precursor in the production of Cu- and REE-containing oxides.

The semi-amorphous nature of synthesised LDH prevents a full structural analysis by standard crystallographic methods. Further work on these materials with the application of synchrotron techniques is in progress.

## Figures and Tables

**Table 1:** Chemical composition of the synthetic samples. The values are expressed in wt %.

Sample	Cu	Al	S	Y	La	Ce	Nd	Gd
LDH 3CuAl·SO_4_-pH8	39.83	5.78	4.79	-	-	-	-	-
LDH 3CuAl·SO_4_-pH10	44.7	3.59	4.50	-	-	-	-	-
LDH-Y-8	40.60	4.50	4.40	3.50	-	-	-	-
LDH-Y-10	41.50	4.60	3.40	3.50	-	-	-	-
LDH-La-8	37.30	3.70	4.90	-	4.90	-	-	-
LDH-La-10	32.70	4.10	6.30	-	5.20	-	-	-
LDH-Ce-8	33.8	4.20	6.40	-	-	3.8	-	-
LDH-Ce-10	42.10	4.90	5.00	-	-	5.10	-	-
LDH-Nd-8	32.9	4.05	6.20	-	-	-	4.4	-
LDH-Nd-10	35.8	2.32	5.60	-	-	-	8.0	-
LDH-Gd-8	36.76	4.34	4.12	-	-	-	-	4.04
LDH-Gd-10	36.30	4.33	5.15	-	-	-	-	4.26

**Table 2:** Chemical and crystallographic characteristics of synthetic LDH.

Sample	Molar Ratio (in Solution)	LDH Formula	x (Al/Cu + Al)	d001 (Å)	2x d002 (Å)	*a* = 2x d110 (Å)
LDH 3CuAl·SO_4_-pH8	3Cu:Al	[Cu_5.92_Al_2.08_(OH)_16_]S_1.04_	0.25	7.90	7.92	3.060
LDH 3CuAl·SO_4_-pH10	3Cu:Al	[Cu_6.40_Al_1.60_(OH)_16_]S_0.80_	0.20	8.17	8.03	3.067
LDH-Y-8	3Cu:0.8Al:0.2Y	[Cu_5.68_(Al_1.84_Y_0.48_)_Σ2.32_(OH)_16_]S_1.16_	0.24	7.88	7.83	3.048
LDH-Y-10	3Cu:0.8Al:0.2Y	[Cu_5.49_(Al_2.03_Y_0.48_)_Σ2.51_(OH)_16_]S_1.255_	0.23	8.03	8.20	3.038
LDH-La-8	3Cu:0.8Al:0.2La	[Cu_6.34_Al_1.66_(OH)_16_]S_0.83_	0.21	7.70	7.61	3.066
LDH-La-10	3Cu:0.8Al:0.2La	[Cu_6.18_Al_1.82_(OH)_16_]S_0.91_	0.23	8.25	8.02	3.053
LDH-Ce-8	3Cu:0.8Al:0.2Ce	[Cu_6.13_(Al_1.79_Ce_0.08_)_Σ1.87_(OH)_16_]S_0.935_	0.23	7.74	7.64	3.067
LDH-Ce-10	3Cu:0.8Al:0.2Ce	[Cu_6.10_Al_1.90_(OH)_16_]S_0.95_	0.24	8.39	8.22	3.082
LDH-Nd-8	3Cu:0.8Al:0.2Nd	[Cu_5.93_(Al_1.72_Nd_0.35_)_Σ2.07_(OH)_16_]S_1.035_	0.22	8.05	7.74	3.050
LDH-Nd-10	3Cu:0.8Al:0.2Nd	[Cu_6.39_(Al_0.98_Nd_0.63_)_Σ1.61_(OH)_16_]S_0.805_	0.13	7.92	-	-
LDH-Gd-8	3Cu:0.8Al:0.2Gd	[Cu_6.05_(Al_1.68_Gd_0.27_)_Σ1.95_(OH)_16_]S_0.975_	0.22	7.78	7.68	3.069
LDH-Gd-10	3Cu:0.8Al:0.2Gd	[Cu_6.02_(Al_1.69_Gd_0.29_)_Σ1.98_(OH)_16_]S_0.99_	0.22	8.06	7.93	3.077
